# A mariner's tale: Invasive endotracheal *Mycobacterium marinum* infection

**DOI:** 10.1002/rcr2.1211

**Published:** 2023-08-31

**Authors:** Peter T. Bell, James Anderson, Christopher Coulter, Andrew J. Dettrick, Andrew Burke, Timothy Baird

**Affiliations:** ^1^ Department of Respiratory Medicine Sunshine Coast University Hospital Birtinya Queensland Australia; ^2^ Faculty of Medicine The University of Queensland Brisbane Queensland Australia; ^3^ Queensland Mycobacterial Reference Laboratory, Pathology Queensland Royal Brisbane Women's Hospital Campus Brisbane Queensland Australia; ^4^ School of Health University of the Sunshine Coast Sunshine Coast Queensland Australia; ^5^ Pathology Queensland Sunshine Coast University Hospital Birtinya Queensland Australia; ^6^ Department of Thoracic Medicine The Prince Charles Hospital Brisbane Queensland Australia; ^7^ University of Queensland Centre for Clinical Research, Faculty of Medicine The University of Queensland Brisbane Queensland Australia

**Keywords:** bronchoscopy, *Mycobacterium marinum*, non‐tuberculous mycobacterium, pulmonary infection, trachea

## Abstract

*Mycobacterium marinum* is a ubiquitous water‐borne non‐tuberculous mycobacterial (NTM) pathogen. In humans, *M. marinum* infections are acquired through direct inoculation of skin wounds and are almost exclusively localized to skin and soft tissues. Pulmonary infection with *M. marinum* is extremely rare, and to our knowledge, invasive endobronchial disease has not been reported. Here, we present a case of a 71‐year‐old immunocompetent male surfer with invasive endotracheal *M. marinum* granulomatous disease. The patient was successfully cured with a regimen of azithromycin 250 mg daily, ethambutol 900 mg (15 mg/kg) daily and rifampicin 600 mg daily for 12 months following culture conversion. This case highlights several important concepts: Firstly, *M. marinum* infection, including invasive endobronchial infection, should be considered a rare cause of NTM pulmonary disease. Secondly, endotracheal infection can be successfully eradicated with this selected therapeutic regimen. Finally, the absence of *M*. *marinum* skin or soft‐tissue infection in this patient, raises the possibility that human disease might also be acquired via inhalation of *M*. *marinum* contaminated water in rare circumstances.

## Graphical abstract

In humans, *Mycobacterium marinum* infections are acquired through direct inoculation of skin wounds and are almost exclusively localized to skin and soft tissues. Pulmonary infection with *M. marinum* is extremely rare, and to our knowledge, invasive endobronchial disease has not been reported previously. Here, we present a case of a 71‐year‐old apparently immunocompetent male surfer with invasive endotracheal M. marinum granulomatous disease. The patient was successfully cured with a 12‐month regimen of azithromycin 250 mg daily, ethambutol 900 mg daily and rifampicin 600 mg daily.

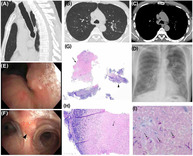

## INTRODUCTION

*Mycobacterium marinum* is a water‐borne non‐tuberculous mycobacteria (NTM) ubiquitously distributed in aquatic environments.^1^ In humans, *M. marinum* causes skin and soft tissue disease, resulting in chronic granulomatous ulcers, nodules and nodular lymphangitis. Infection occurs via direct inoculation, when contaminated water is exposed to skin wounds or abrasions. While contemporary chlorination practices have reduced *M. marinum* outbreaks in public swimming pools,^2^ *M. marinum* continues to be an important cause of human skin infection in individuals who engage in natural outdoor marine activities or cleaning and maintenance of fish tanks.^1^

In humans, *M. marinum* infections are nearly always localized to skin and soft tissues, in part due to the portal of inoculation and the growth characteristics of the organism; which demonstrates optimal growth at 30°C and suboptimal replication at 37°C.^1^, ^2^ In some cases*, M. marinum* infection results in locally invasive disease into deeper soft‐tissue structures, including tenosynovitis, osteomyelitis, arthritis, and bursitis.^2^ Rare cases of disseminated infection have been reported in severely immunocompromised patients on anti‐TNF therapy or following solid‐organ/haematopoietic stem cell transplantation.^1^ Pulmonary infection with *M. marinum* is extremely rare; with only five cases in apparently ‘immunocompetent’ individuals reported in the literature to date.^1^, ^3^, ^4^, ^5^, ^6^ Invasive endobronchial *M. marinum* infection has never been reported to our knowledge.

## CASE REPORT

**FIGURE 1:**
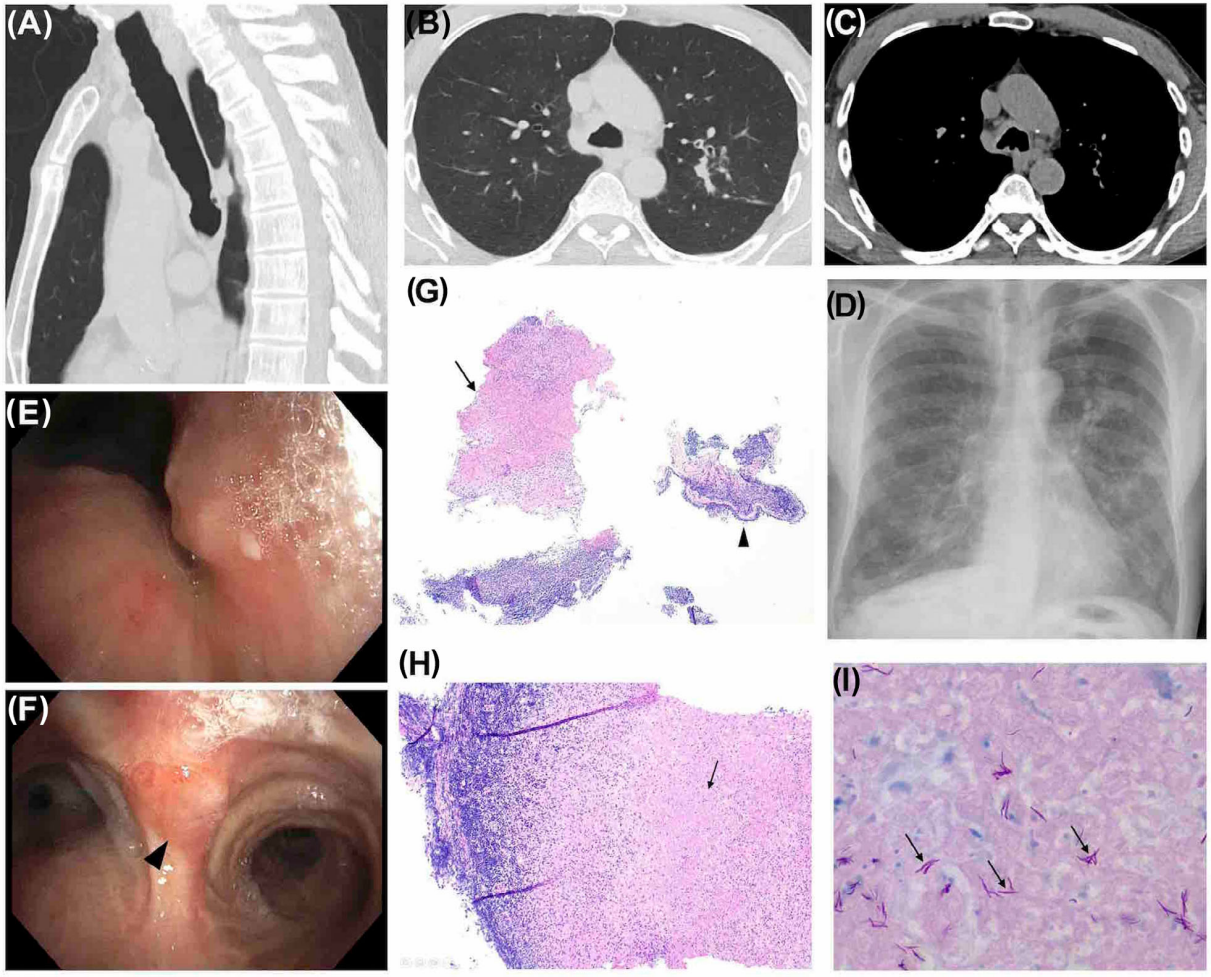
(A). Sagittal view of endotracheal soft tissue lesion with invasion into distal tracheal wall. (B) Axial view demonstrates left upper lobe bronchiectasis characterized by bronchial wall thickening and mucus plugging. (C) Axial view (mediastinal windows) demonstrate an absence of significant mediastinal or hilar lymphadenopathy or calcification. (D) Posteroanterior (PA) plain film demonstrates upper lobe nodular infiltrates. (E) White light bronchoscopy of an abnormal endotracheal lesion affecting the lateral wall of the distal trachea. (F) White‐light bronchoscopy demonstrates an abnormal endotracheal lesion affecting the distal trachea/carina which was biopsied (biopsy site demarcated by black arrow). (G) Histopathology of endobronchial biopsy fragments showing inflammation and caseous necrosis (arrow) (H&E, original mag. ×40). (H) High‐power showing bronchial mucosa with granulomatous inflammation with caseous necrosis (arrow) (H&E, original mag. ×100). (I) Copious acid‐fast bacilli (arrows) in the necrosis (Ziehl‐Neelson, original mag. ×1000).

A 71‐year‐old Caucasian male never a smoker was referred to our service for investigation and management of suspected NTM pulmonary disease. The patient had a 24‐month history of productive cough with tenacious purulent sputum, mild left upper lobe nodular infiltrates, cylindrical bronchiectasis and an endoluminal soft tissue irregularity of the distal trachea on thoracic Computed Tomography (Figure 1A–D). Acid‐fast bacilli had been identified on Ziehl–Neelsen staining and *M. marinum* had cultured from an expectorated sputum sample.

Medical history included childhood‐onset allergic asthma with moderately‐severe fixed expiratory airflow limitation (FEV_1_ 1.57 L, 55.1% predicted), allergic rhinosinusitis, varicella zoster virus reactivation, gastroesophageal reflux disease and keratoacanthomas. Medications were inhaled fluticasone propionate/salmeterol xinafoate (200/6 mcg) 2 puffs daily, pantoprazole 40 mg daily, intranasal mometasone 50 mcg spray daily. He was prescribed 1–2 short courses of oral prednisone (25 mg for 5–7 days) most winters in recent years for poorly controlled asthma.

The patient had worked as a carpenter for the majority of his adult‐life, and now enjoys an active‐lifestyle in retirement. He spends several hours every day surfing the beaches of the Sunshine Coast, Queensland, Australia. In previous years, he had travelled to Samoa and Indonesia on surfing trips. On his most recent trip to Indonesia 4 years ago he suffered a minor superficial lower limb laceration on coral which healed without medical intervention. The patient's hobbies also included renovating an old yacht which was located in a nearby shipyard. In the past, he had cleaned the underside of the boat with a high pressure hose, sanded down the timber surfaces and reinforced them with fibreglass. He lives with his partner on a small ‘hobby‐farm’ in the Sunshine Coast hinterland. They drink filtered tank rainwater from the property and have horses and pigs on the property. His father suffered *Mycobacterium tuberculosis* pulmonary infection 40 years ago. There was no other family history of mycobacterial infection, bronchiectasis or immunodeficiency. Physical examination was unrevealing.

Bronchoscopy revealed thick tenacious purulent sections throughout the trachea and main bronchi. Foci of abnormal mucosa affecting the lateral wall of the distal trachea (Figure 1E) and the carina (Figure 1F, biopsied) were identified. Severe stricturing of the proximal left upper lobe bronchus did not allow advancement of the bronchoscope into the left upper lobe. Histopathological examination of the biopsied distal tracheal mucosal lesion revealed necrotising granulomatous inflammation with copious mycobacteria seen on Ziehl–Neelsen and Wade Fite stains (Figure 1G–I). Bronchial washings demonstrated growth of mycobacteria in liquid culture medium (BACTEC MGIT 960) at 36°C after 17 days of incubation. The identity of the cultured photochromogenic mycobacterial species was confirmed as *M. marinum* by hsp65 and 16S rRNA gene sequencing. Macrolide‐susceptible *M. marinum*, as well as copathogens *Serratia marcescens and Burkholderia gladioli*, were cultured. Comprehensive screening did not identify a known immunodeficiency syndrome or alternate causes for bronchiectasis (Table 1). Mycobacterial blood cultures were negative. There was no evidence of occult deep‐seated soft tissue infection on fluorodeoxyglucose Position Emission Tomography. Mycobacterial culture of environmental sample from his home drinking rain tank water at time of diagnosis did not culture mycobacteria. Functional autoantibodies to interferons demonstrated normal signal transducer and activator of transcription 1 (STAT1) phosphorylation in response to interferon (IFN)‐alpha, IFN‐gamma, and IFN‐omega in the presence of patient serum.

**TABLE 1:** Bronchiectasis & immunodeficiency screen.

Assay	Result	Normal range
CFTR genotype	No pathogenic variant	–
IgG	7.4	7.0–16.0 g/L
IgA	3.8	1.0–4.0 g/L
IgM	0.6	0.4–2.3 g/L
IgE	264	kU/L
ANA	1:160 (Homogenous)	<40
ENA	Negative	–
Anti‐CCP	2	<6 U/mL
RF	<20	<20
HIV	Negative	–
C3	1.38	0.90–1.80 g/L
C4	0.46	0.10–0.40 g/L
CH50	805	>520 U/mL
Lymphocyte subset	No abnormality	–
Peripheral flow cytometry	No abnormality	–
FLC	1.05	0.31–1.56 mg/L
MBL	>3000 ng/mL	>1300 ng/mL
SPEP	No abnormality	–
NOB	288	>70 SI (MFI)
Anti‐IFN gamma antibodies	Normal response	–

Abbreviations: ANA, anti‐nuclear antibodies; Anti‐CCP, anti‐cyclic citrullinated peptide antibodies; C3, C3‐Complement; C4, C4, C4‐Complement; CH50, CH50 total complement activity; CFTR, cystic fibrosis transmembrane regulator; ENA, extractable nuclear antibodies; FLC, free light chains; HIV, human immunodeficiency virus; Ig, immunoglobulin; IFN, interferon; MBL, Mannose binding lectin; NOB, neutrophil oxidative burst test; RF, rheumatoid factor; SPEP, serum protein electrophoresis.

**FIGURE 2:**
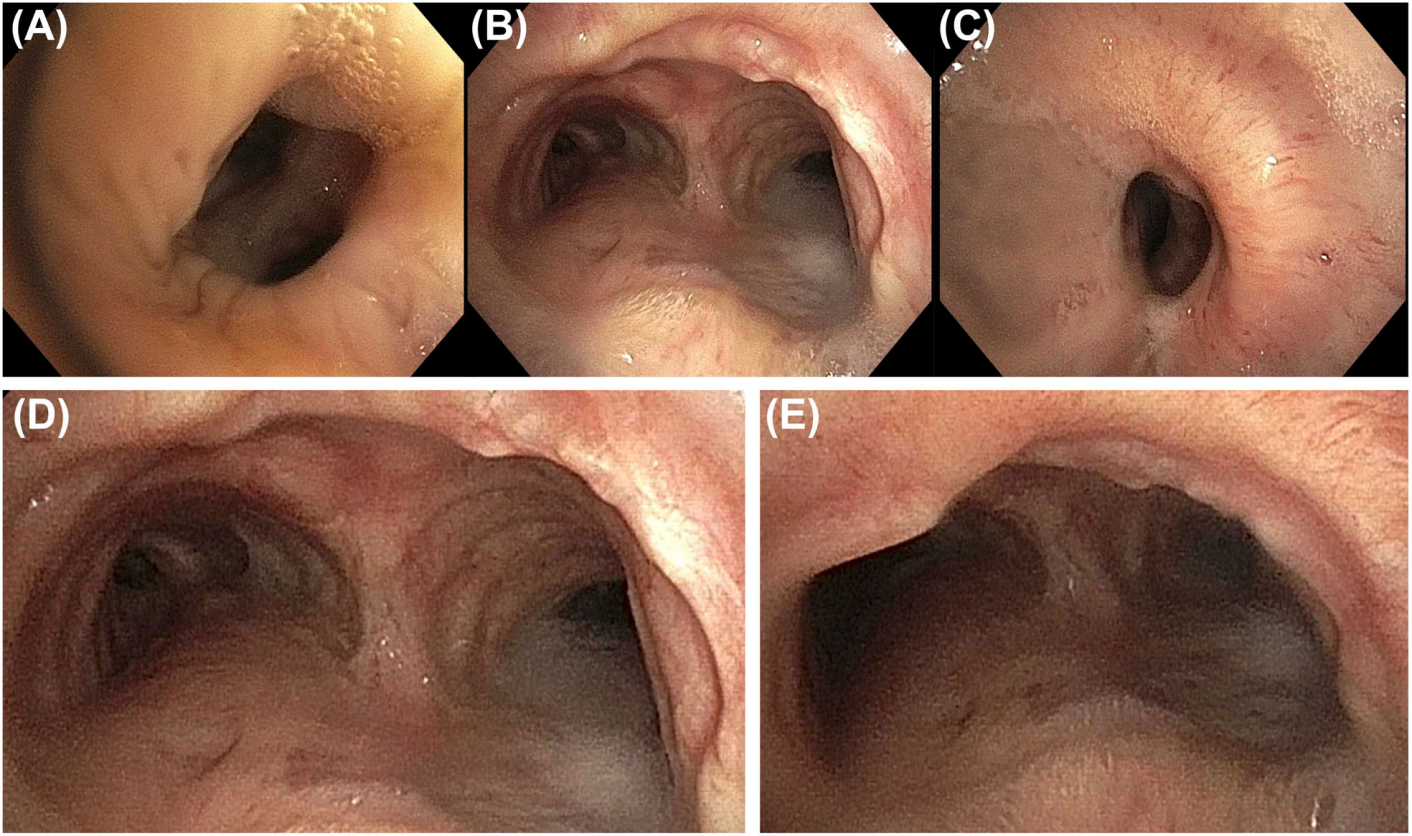
Twelve‐months of treatment after culture conversion, white‐light bronchoscopy demonstrated near‐complete resolution in macroscopic endotracheal abnormalities. (A/C) Relative improvement in the patency of the left upper lobe bronchus but with a degree of persistent bronchostenosis at the upper lobe bifurcation and left upper lobe bronchus proper. (B/D) Resolution of the mucosal lesion previously seen at the location of the carina (this was the location originally biopsied and demonstrated in Figure 1F prior to commencement of therapy). (E) Resolution of the erythematous mucosal protuberance affecting the lateral wall of the distal trachea.

Endotracheal *M. marinum* infection was treated with azithromycin 250 mg daily, ethambutol 900 mg daily (15 mg/kg) and rifampicin 600 mg daily, for a total of 12 months after bronchoscopic culture conversion. *S. marcescens and B. gladioli* coinfections were also treated with a 14‐day course of trimethoprim‐sulfamethoxazole at treatment outset. At 3‐month follow‐up, there was a complete resolution of clinical symptoms and the patient returned to surfing in the ocean. Clinical improvement was also associated with complete resolution of bronchial secretions with smear and culture conversion from bronchoscopy washings. After 12‐months of treatment, white‐light bronchoscopy demonstrated near‐complete resolution in macroscopic endotracheal abnormalities; with resolution of the erythematous mucosal protuberance affecting the lateral wall of the distal trachea (Figure 2E), as well as the mucosal lesion previously seen at the location of the carina (Figure 2B/D). There was also relative improvement in the patency of the left upper lobe bronchus, but with a degree of persistent bronchostenosis at this location (Figure 2A/C). There were minor thin secretions in the trachea alone. Washings from the main airways and left upper lobe were smear negative on Ziehl–Neelsen and Wade Fite stains. Specimens were negative on bacterial and mycobacterial culture. Twelve months after bronchoscopic culture conversion (i.e., 15‐months total treatment duration) therapy was ceased and the patient remains under follow‐up surveillance.

## DISCUSSION

To our knowledge, this is the first reported case of invasive endobronchial *M*. *marinum* disease and remains one of the only ever reported cases of human *M*. *marinum* pulmonary infection in an apparently immunocompetent host.^1^, ^3^, ^4^, ^5^ This rare case highlights several important points. First, *M. marinum* infection, including invasive endobronchial infection, should be considered a rare cause of granulomatous pulmonary disease in patients with epidemiological risk factors for acquisition, such as individuals with exposure to fish or environmental water habitats. Second, *M. marinum* pulmonary infection can occur, albeit very rarely, in patients without identifiable immunodeficiency despite comprehensive evaluation. Third, invasive endotracheal *M. marinum* infection can be successfully treated with a regimen of azithromycin 250 mg, ethambutol 15 mg/kg and rifampicin 600 mg daily. Finally, the absence of *M*. *marinum* skin or soft‐tissue infection, raises the possibility that human disease might also be acquired via *M*. *marinum* inhalation of contaminated water.

*M. marinum* cutaneous and soft tissue infections are usually treated with antibiotics with or without surgical debridement. In the absence of clinical trial data; the choice, combination and duration of antibiotic therapy in this context remains uncertain. Even greater uncertainty clouds the clinical management of *M. marinum* pulmonary disease. In this case, we successfully utilized a treatment regimen of azithromycin, ethambutol and rifampicin for a duration of 12 months following culture conversion; an approach extrapolated from expert consensus guidelines on the management of *Mycobacterium avium* complex (MAC) pulmonary disease.^7^ This regimen was associated with early clinical response and ultimately disease cure.

It should be recognized that although this patient did not have a detectable immunodeficiency syndrome; asthma, inhaled corticosteroids use, intermittent oral steroids and gastroesophageal reflux disease are known host risk factors non‐tuberculous mycobacterial infection.^8^ In addition, while we cannot completely exclude contribution of *S. marcescens and B. gladioli* coinfection in this clinical presentation; the histological finding of abundant mycobacteria associated with caseating granulomas suggests *M. marinum* as the causative pathogen.

Although *M. marinum* demonstrates optimal growth at 30*°*C and more limited replication at 37°C, we cultured the pathogen on liquid culture media at 36*°*C after 17 days, demonstrating the ability of the isolate to replicate at temperature ranges present at the distal tracheal mucosal interface.^9^ This raises the possibility that incidence of pulmonary *M. marinum* infection has been underestimated as incubation at the optimal growth temperature of 30*°*C is not usually utilized for respiratory samples (as opposed to skin and soft tissue specimens where incubation at physiological and a lower temperature is routine).

This case expands the spectrum of human *M. marinum* infection to include invasive endobronchial granulomatous disease which is treatable using a three‐drug combination antibiotic regimen.

## AUTHOR CONTRIBUTIONS

All authors contributed to drafting and editing of the manuscript.

## CONFLICT OF INTEREST STATEMENT

None declared.

## ETHICS STATEMENT

The authors declare that appropriate written informed consent was obtained for the publication of this manuscript and accompanying images.

## Data Availability

The data that support the findings of this study are available on request from the corresponding author. The data are not publicly available due to privacy or ethical restrictions.
